# Providers of and participants in suicide assistance in Germany: A field description based on expert interviews

**DOI:** 10.1371/journal.pone.0350926

**Published:** 2026-06-18

**Authors:** Sven Schwabe, Franziska A. Herbst

**Affiliations:** Institute for General Practice and Palliative Care, Hannover Medical School, Hannover, Germany; University of Foggia: Universita degli Studi di Foggia, ITALY

## Abstract

**Background:**

Over the past 20 years, assisted dying practices have been legalised in an increasing number of countries around the world. After a decision of the German Federal Constitutional Court in 2020, suicide assistance is unregulated in Germany but official data about the implementation of suicide assistance is lacking.

**Aim:**

This study aims to explore the providers of and participants in suicide assistance in Germany.

**Methods:**

16 semi-structured qualitative expert interviews were conducted with scientists, officials, journalists and members of right-to-die organisations between May and July 2025. Experts were chosen due to their extensive theoretical and practical knowledge of the practice of assisted suicide in Germany. The interviews were digitally recorded, transcribed verbatim and analysed using qualitative content analysis, according to Mayring.

**Results:**

A total of five main providers and participants involved in the practice of assisted suicide in Germany were identified: 1) right-to-die organisations, 2) medical and non-medical suicide assistants, 3) treating physicians, 4) funeral directors and 5) relatives and friends. Right-to-die organisations and suicide assistants from various professions regularly provide suicide assistance and carry out all relevant tasks, including the provision of information, organisation, implementation and documentation. Treating physicians, funeral directors, relatives and friends may be asked to provide suicide assistance in individual cases and take on specific tasks. Other healthcare professionals (e.g., nurses, pharmacists) and professional groups (e.g., police officers, prosecutors) may also be confronted with suicide assistance and become involved in the process.

**Discussion:**

Given the minimal regulation of assisted suicide in Germany, the landscape of providers and supporters appears to be diversifying and growing increasingly complex. Future research should examine the roles, experiences, challenges and best practices of the various actors involved in the provision of assisted suicide.

**Trial registration:**

The study was prospectively registered in the German Clinical Trials Register (Deutsches Register Klinischer Studien) under Registration N° DRKS00034574, on 04 July 2024. It is also searchable via the International Clinical Trials Registry Platform Search Portal of the World Health Organization, under the same registration number.

## Introduction

On 26 February 2020, the Federal Constitutional Court of Germany declared the ban on repeated suicide assistance unconstitutional and repealed Section 217 of the German Criminal Code, which had only been introduced in 2015 [1]. Attempts to re-regulate assisted suicide in 2023 were unsuccessful. As a result, assisted suicide has remained unregulated in Germany for 6 years [2].

Assisted suicide is defined as the act of providing an individual with the means to end their own life, such as by supplying them with lethal medication. The individual seeking suicide self-administers the medication, either by ingestion or, in the case of intravenous infusion, by manually triggering the substance delivery. In all instances, the individual wishing to die remains the primary agent in the act. Thus, it is distinct from killing on demand (in international debate: euthanasia), in which a third party assumes control over the act. Killing on demand is illegal in Germany under Section 216 of the Criminal Code [3]. This tendency in Germany towards the liberalisation of assisted dying is in line with developments in other European countries in recent years. Following the long-standing legalisation of assisted suicide in Switzerland and of assisted suicide and euthanasia in the Netherlands and Belgium, a number of other countries have also introduced legislation regulating assisted suicide and, in some cases, euthanasia since 2019 [4]. These countries include Italy (2019) [5], Austria (2020) [6], Spain (2021) [7], and France (2025) [8].

In Germany the 2020 ruling by the Federal Constitutional Court has reignited the debate on assisted suicide. Medical associations have removed the ban on physicians’ involvement in assisted suicide from their professional codes of conduct [9], associations and professional societies have published discussion and position papers on the subject [10–12] and numerous articles and case reports on assisted suicide have appeared in the media. According to self-reported data from right-to-die organisations, the number of assisted suicides carried out by these organisations rose rapidly from fewer than 100 in 2020 to more than 1,000 in 2024 [13]. This steady increase mirrors trends observed in other countries, such as Canada, where the proportion of assisted suicides has risen continuously since legalisation, reaching 4.7% of deaths in 2024 [14]. According to the available data, assisted suicides currently account for 0.1% of all deaths in Germany. However, since assisted suicide remains a taboo subject in Germany, and there is no nationwide reporting system, the number of unreported cases is likely high.

An international scoping review by Fujioka et al. [15] demonstrated that, depending on the legal context, various actors are involved in the organisation and implementation of assisted suicide, each assuming different tasks and responsibilities. Physicians appear to play a pivotal role, participating in all aspects of the process, including the initial consultation, counselling, provision of medication, administration and monitoring of the procedure, and documentation [16–18]. In the international context, nurses also play a key role, providing counselling to those considering suicide and to other healthcare professionals, and, in certain situations, administering medication and offering bereavement support [19,20]. Furthermore, the involvement of social workers, pharmacists and psychologists at various stages of the assisted suicide process has been documented [21–24]. Finally, relatives frequently wish to be included from the outset, so they have time to prepare for the impending loss of a family member and to ensure access to professional support [25].

Scientific information on assisted suicide in Germany remains limited and is only available for certain regions. An evaluation of death certificates in the Munich area shows that 37 assisted suicides took place between 2020 and 2022, of which 36 were accompanied by right-to-die organisations [26]. According to the report, those present during the assisted suicides were primarily physicians and employees of three different right-to-die organisations, as well as lawyers and relatives [27].

The proportion of healthcare professionals in Germany who express willingness to personally assist in assisted suicide — regardless of the health status of the individuals concerned — ranges from 13.9% to 20.9%, while 3% report having already provided such assistance [28–30]. A qualitative interview study from Germany also found that general practitioners are regularly asked to assist with suicide and that some do so frequently [31]. Since November 2024, requests for and instances of assisted suicide in Germany have been documented through self-reporting in a dedicated registry. However, the number of recorded cases remains low [32]. At present, there is no comprehensive understanding of the individuals, professional groups and organisations involved in the practice of assisted suicide, or their specific roles. This gap in knowledge is particularly concerning, given that assisted suicide is not yet subject to legal regulation in Germany.

### Study aim

The present study aimed to explore the providers of suicide assistance and participants in this process in Germany. The research question is: Which providers and participants of assisted suicides in Germany exist and which tasks do they perform? In this manuscript, providers and participants refer to individuals who directly and indirectly, respectively, contribute to facilitating assisted suicide.

## Materials and methods

### Design

The ASEP-study was a prospective, observational qualitative investigation that explored and analysed the practice of assisted suicide in Germany following an interpretative approach [33]. Qualitative guideline-based interviews were conducted with experts on assisted suicide in Germany to identify the providers and participants involved in its current practice. The methodology and reporting followed the Consolidated Criteria for Reporting Qualitative Research (COREQ) [34].

### Interview guideline

The interview guide (see S1 File. Interview guide) was developed by the authors on the basis of the research literature. Topics for the guide were derived from the scoping review conducted by Fujioka et al. [15]. The guide was designed to elicit information about providers and participants, as well as their specific activities and the challenges associated with the provision of suicide assistance in Germany. The present manuscript specifically explores the providers and participants involved in this practice, whereas findings related to their activities and challenges will be presented elsewhere. Each topic area was introduced with an open-ended, narrative-generating question, followed by more detailed enquiries. The effectiveness of the guide was evaluated through two pilot interviews, after which revisions were made in accordance with interviewee feedback. In addition, a brief questionnaire was developed and approved for collecting socio-demographic data from participants (see S2 File. Sociodemographic questionnaire).

### Sampling

Interviewees were selected on the basis of their professional and organisational activities, which provided them with extensive theoretical and practical knowledge of the practice of assisted suicide in Germany. Theoretical knowledge refers to scientific research or professional engagement with the topic, while practical knowledge indicates repeated organisational or hands-on involvement in assisted suicide in Germany. All interviewees were required to be at least 18 years of age and to provide written consent for participation. Interviewees were purposively selected according to the above criteria. The number of planned expert interviews was initially set at *n* = 10. However, as interviewees recommended further experts, the sample was expanded using snowball sampling, with the aim of obtaining a more comprehensive picture of the research topic. The interview study was concluded once all identified interviewees had been interviewed and no further interviews revealed any additional providers, participants or interviewees.

### Recruitment

In April and May 2025, scientific societies, right-to-die organisations and researchers working in the field of assisted suicide were requested via telephone and email to provide assistance in the identification of suitable interview partners. Initial interview requests were sent to the German Association for Suicide Prevention, the German Association for Palliative Medicine, the German Hospice and Palliative Care Association, the German Medical Association, the Research Network of the German Research Foundation (DFG) ‘Scientific Foundation for a Responsible Practice of Assisted Suicide. An Interdisciplinary Network to Investigate Normative and Empirical Requirements’ and three German right-to-die organisations (the German Society for Human Dying, Dignitas Germany and Sterbehilfe Germany). Additional individuals (e.g., journalists) and a fourth right-to-die organisation were included via snowball sampling. The experts identified through this process were subsequently contacted individually via email and invited to participate in an expert interview. Where multiple recommendations were received for a single association or professional body, contact was initially made with a single representative from the respective group. Upon receiving a positive response, interview appointments were arranged by telephone or email. Participants were also provided with detailed information about the study, a declaration of consent, a brief socio-demographic questionnaire and a reimbursement form for expenses amounting to €50.

### Data collection

A total of 24 experts were invited to participate in the interviews. Depending on their preferences, the interviews were conducted in person, by telephone or via video conference using Microsoft Teams. In one instance, Webex video conferencing software was used, for compatibility reasons. Prior to conducting the interviews, the researchers had not established any form of relationship with the interviewees. All interviews were conducted by the first author, who is a trained sociologist with a rigorous training in qualitative and quantitative methods of empirical social research and extensive experience in qualitative social research. The interviewer had no personal, commercial or political interest in the research topic. The interviews commenced with an introductory question. This enabled the participants to introduce themselves and establish a rapport with the interviewer. If an interviewee did not understand a question, it was repeated using different words. If an interviewee did not wish to answer a question, it was omitted. During the interviews, care was taken to adapt the order and phrasing of the questions in accordance with the general flow of the conversation, rather than adhering rigidly to ‘guideline bureaucracy’ [35]. The interviews were digitally recorded, transcribed verbatim and pseudonymised. None of the participants requested to have their transcripts returned. Participants were not asked to comment on the results of the study.

### Data analysis

The pseudonymised interview transcripts were analysed using qualitative content analysis as outlined by Mayring [36], with the support of MAXQDA2024 software. In a deductive coding process, the first author initially assigned all text passages – referring to providers and participants of suicide assistance – to the predefined categories from the interview guide and documented emerging ideas and assumptions in code-specific memos. Where providers or participants could not be allocated to an existing category, new codes were generated inductively. Some categories of providers and participants were derived deductively from the literature (e.g., right-to-die organisations, physicians, relatives and friends, nurses), whilst others were developed inductively from the data. In some cases, deductive categories were refined on the basis of the data (e.g., treating physicians and physician suicide assistants). In a second step, the activities of the respective providers and participants were summarised and coded within the main categories through an inductive coding process (Table 1). Coding was randomly reviewed by a second member of the research team. Any discrepancies were discussed, and the coding framework was subsequently refined.

**Table 1:** Code Tree – Providers of and Participants in Suicide Assistance.

Category	Code
Right-to-die organisations	- Information and advice - Assessment of the desire to die - Organisation of suicide assistance - Involvement of relatives and friends - Accompanying suicide assistance - Documentation
Suicide assistants - Physician suicide assistants - Non-physician suicide assistants	- Information and advice - Assessment of the desire to die - Provision of medication - Involvement of relatives and friends - Accompanying suicide assistance - Documentation - Death certificate and post-mortem examination - Information and advice - Assessment of the desire to die - Organisation of suicide assistance - Involvement of relatives and friends - Accompanying suicide assistance - Documentation
Treating physicians	- Information and advice - Assessment of the desire to die - Organisation of suicide assistance - Provision of medication - Involvement of relatives and friends - Accompanying suicide assistance - Death certificate and post-mortem examination
Funeral directors	- Information and advice - Organisation of suicide assistance - Removal of the corpse
Relatives and friends	- Information and advice - Organisation of suicide assistance - Accompanying suicide assistance
Indirectly involved players	- Emergency service - Pharmacies - Nurses - Ethical consulting teams - Pastoral caregivers - Hospice care volunteers - Specialist palliative home care teams - Police - Public prosecutors

### Ethics statement

Ethics approval and consent to participate: This study was performed in accordance with the declaration of Helsinki and approved by the Ethics Committee of Hannover Medical School (N° 10196_BO_S_2022) on 22 August 2023.

### Informed consent

Individual written informed consent to participate was obtained in writing from all participants. Individual written informed consent for the research team to publish the study results was obtained from all participants.

## Results

### Sample characteristics

Of the 24 individuals invited to participate, 16 provided consent and ultimately took part. Reasons for non-participation included non-response (*n* = 4), lack of expertise (*n* = 3) and lack of time (*n* = 1). Interviews lasted an average of 52 minutes (range: 29–90 minutes), and the mean age of participants was 52.3 years (range: 32–70 years). Fourteen participants were female, and more than half (*n* = 9) held a PhD as their highest educational qualification (Table 2).

**Table 2:** Sample Socio-Demographic Characteristics.

		*n*	%
No. participating experts		16	100
Sex	Female	14	88
	Male	2	13*
Age	31–45 years	5	31
	46–60 years	8	50
	>60 years	3	19
Highest educational level	PhD	9	56
	Master’s	6	38
	Vocational training	1	6
Professional background (multiple answers possible)	Medicine	5	29
	Nursing/nursing science	4	24
	Humanities/social sciences	2	12
	Psychology	2	12
	Law	1	6
	Journalism	1	6
	Philosophy/ethics	1	6
	Various	1	6
Field of activity (multiple answers possible)	Research/academia	9	47
	(Expert) Association	3	16
	Patient care	3	16
	Other	4	25
Direct participation in suicide assistance	Yes	5	31
	No	11	59
Years working on assisted suicide	≤5	8	50
	6–10	2	13
	11–15	3	19
	>15	3	19

Note. *Rounding differences may occur

### Interview findings

**Fig 1:**
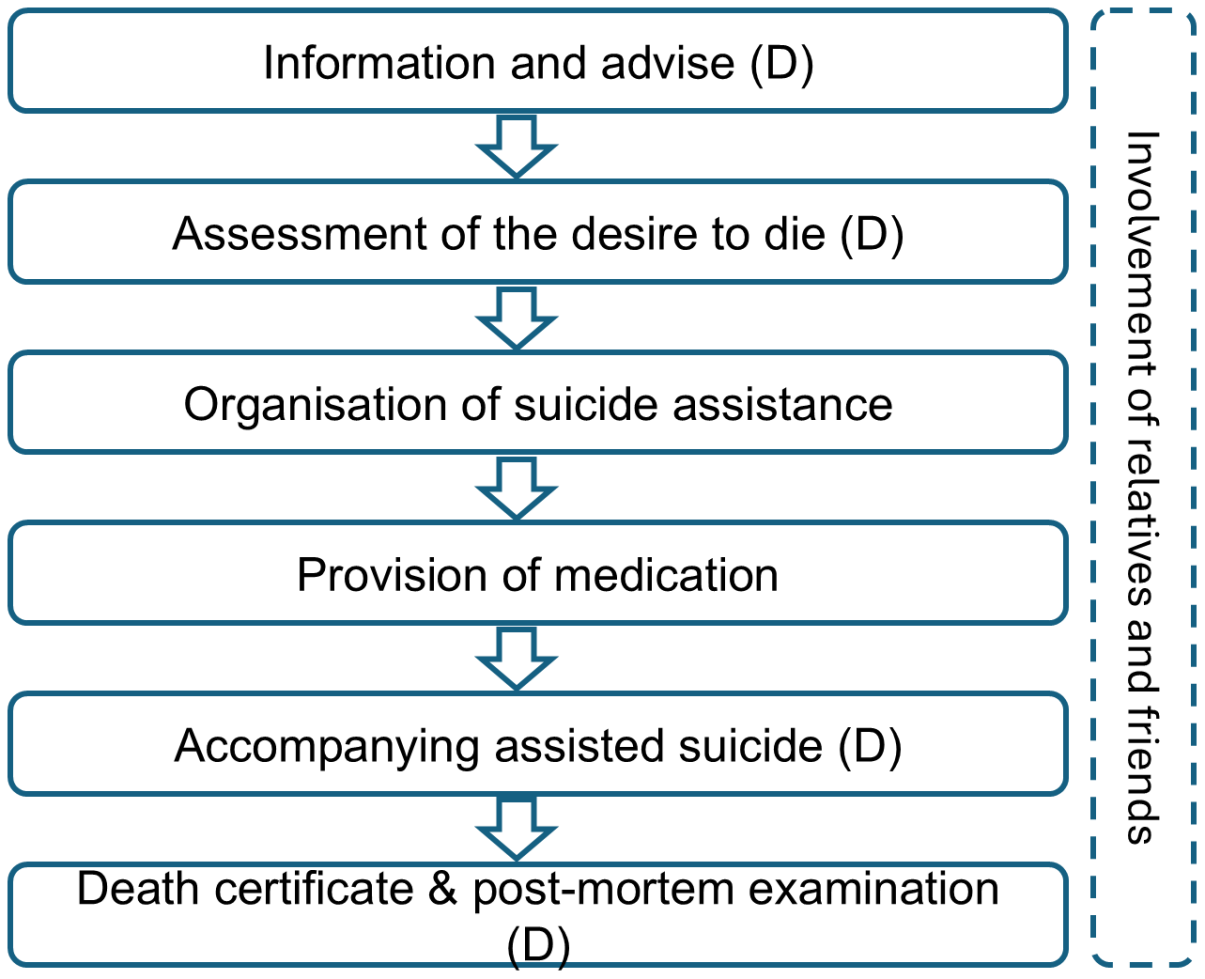
Schematic overview of the assisted suicide process. **(D)**: documentation.

Analysis of the interviews revealed five main categories of providers and participants involved in the practice of assisted suicide in Germany, including right-to-die organisations as institutional providers and a number of further participants with indirect involvement. The following section outlines the main actors and their respective areas of activity, illustrated by interview excerpts. Findings derived exclusively from individuals who had not yet been directly involved in assisted suicide are expressly mentioned. Based on the findings, an initial outline of the assisted suicide process can be reconstructed (Fig 1).

### Right-to-die organisations

A total of four nationwide organisations were mentioned in the interviews as providing information and advice on assisted suicide (to varying degrees and in different ways) and organising assisted suicides. Right-to-die organisations were described as a key source of information on assisted suicide, both for those contemplating suicide and for healthcare professionals.

The right-to-die organisations probably have the most information and offer consultations or information events. They also send out brochures. So I would say that they are the main point of contact for this topic. (I24: 19)

As noted in the interviews, right-to-die organisations have established various procedures and documents to assess, in individual cases, whether the conditions for assisted suicide are met. These procedures generally include a review of documentation (e.g., completed questionnaires, medical records, letters of explanation) and at least one personal conversation with the suicidal individual prior to the assisted suicide. Interview participants reported that this review is conducted by a minimum of two individuals, one of whom must be a medical practitioner. As far es the interviewees were aware, specific time intervals between the request and the performance of assisted suicide are defined.

Various expert opinions are obtained and various discussions are held. An assessment is then made as to whether assisted suicide can be carried out. A certain period of time must elapse before this can be done, to ensure that the wish [to die] is stable. (I24: 09)

The interviews also indicated that right-to-die organisations provide templates for the documentation of assisted suicide (e.g., progress log, waiver of guarantee obligation), facilitate contact with suicide assistants and organise payments (and, if necessary, premises). Moreover, they collaborate with both medical and non-medical suicide assistants, involving them in the organisation of assisted suicides. Interviewees who had not themselves been involved directly in assisted suicide also stated that, in many cases, employees of the organisations themselves act as suicide assistants. They document the procedure, provide support to the suicide assistants and notify the police following the death.

Some right-to-die organisations make a video recording of the process so that they can prove that the person concerned turned the wheel themselves. (I25: 59)

### Suicide assistants

In this work, we define suicide assistants as individuals who regularly provide suicide assistance to a heterogeneous group of individuals. While suicide assistants may come from a range of professions, in practice, they are often physicians or healthcare professionals with experience in patient care (e.g., administering infusions) and access to medication. The quote below illustrates that suicide assistants may be subdivided into physician and non-physician groups.

These are different professional groups, [...] I had met this nurse, then person A, a retired teacher, and there is a doctor who now works as a hypnotist [...]. He also provides suicide assistance. It is often physicians, some of them retired, who do this because they have the time and are perhaps looking for a new field of work. (I9: 126–127)

The interviews indicated that many suicide assistants – particularly physicians – frequently cooperate with right-to-die organisations. Such individuals may be registered in the organisations’ databases and contacted as needed.

It’s a network that has grown. It wasn’t as big at the beginning as it is now, and we’re always on the lookout for new people. Right now, we have a young doctor who is interested in what we do and he is just starting with us. It’s always nice when our network expands. (I21: 130–131)

Depending on the location of the assisted suicide, registered suicide assistants are contacted accordingly. Thus, the number of assisted suicides carried out per assistant may vary considerably. Suicide assistants working within right-to-die organisations are required to adhere to standardised procedures, including initial consultations, assessment of the wish to die and the individual’s free will, compliance with waiting periods, and documentation. Interviewees who had not themselves been involved directly in assisted suicide also mentioned that there are also some suicide assistants, who provide their services independently of such organisations:

There are people who assist in dying outside of right-to-die organisations. For example, doctor A, he used to work for a right-to-die organisation in former times, but they turned their back on him, because he simply didn’t follow their standards. (I25: 13)

When assisted suicide occurs outside of right-to-die organisations, counselling and assessment of free will, the procedure appears to vary significantly, as explained by one interviewee.

I know of individual physicians who do that [suicide assistance] somehow. But as far as I understand, there is no standard procedure or anything like that that they follow. (I11: 9)

Suicide assistants are responsible for procuring the necessary medication, administering the infusion (if required), remaining present during the assisted suicide and documenting the process. Subsequently, they inform the police and hand over the relevant documents. In some instances, the assisting physician also performs the post-mortem examination.

He [the physician who provides suicide assistance] drives there [to the individual wishing to commit suicide] with the drugs, which he obtains through a private prescription, provides assistance and very often also performs the post-mortem examination. (I19: 22)

### Treating practitioners

According to the interviewees, individual general practitioners and specialists (e.g., palliative care physicians, oncologists) sometimes facilitate assisted suicide, particularly when patients frame their request to die as part of their ongoing medical treatment.

There was one case, where death was foreseeable and the family doctor, who knew the patient well, provided assistance in suicide. (I19: 117)

Interviewees noted that treating physicians seldom offer information or advice on assisted suicide proactively.

Family doctors can also provide advice, but only if they have made a conscious decision to deal with the issue and have an open attitude towards it. (I4: 38)

Nevertheless, in individual cases, physicians do provide advice to patients regarding assisted suicide and support them in identifying and organising suicide assistants. Some interviewees, who had not themselves been involved directly in assisted suicide also referred to palliative care physicians who incorporate assisted suicide into their end-of-life care practices.

I know that there is a palliative care network in region A, which I believe is open to providing suicide assistance – on their own or through the involvement of external suicide assistants, I don’t know exactly. But it does exist. Or there is an organisation in region B. I believe they are palliative care physicians who are also open to assisted suicide. There are a few of them. (I9: 146)

Furthermore, respondents indicated that physicians can obtain information on how to conduct assisted suicide by contacting right-to-die organisations. These organisations may share their expertise and, in some cases, invite physicians to observe an assisted suicide procedure. Attending physicians may also provide the necessary medication, prepare it for administration and, depending on the method used, be present during the suicide.

Following the death, a post-mortem examination must be conducted to determine whether the death was unnatural. The issuing of the death certificate and the post-mortem examination are regulated at the federal state level. In many cases, a general practitioner carries out these duties, regardless of whether they were involved in the assisted suicide.

That varies greatly because it is regulated differently in each federal state. In the best case scenario, members have a family doctor, some of whom they have had for many, many years. We can then call the family doctor, inform them, and they will come by during the course of the day. They will then issue the death certificate or confirm the death. (I21: 116)

### Relatives and friends

Interviewees reported that relatives and friends of individuals seeking assisted suicide often find themselves in a position of ambivalence.

On the one hand, ‘I don’t really want to do that because, of course, I don’t want the person to die, but on the other hand, I also want to help somehow because without my help, they might not be able to manage’. And that is often the dilemma they find themselves in. (I24: 41)

The extent to which they are involved in assisted suicide varies considerably, depending on both the wishes of the individual seeking to die and their willingness to participate.

I know that it varies greatly, that there are people who involve their families and who know the exact day and time, and may even be there. And others who know that it is being considered or that the decision has been made, but who are not told the date by the suicidal person. It varies, and there are families who know nothing at all. (I22: 41)

Interviewees described instances in which suicidal individuals asked their relatives or friends to obtain information about suicide assistance or to contact right-to-die organisations and suicide assistants on their behalf. Relatives and friends may also be asked to assist practically in preparing and organising the assisted suicide, particularly when the individual is no longer physically able to manage these tasks independently.

If the person seeking assisted suicide is elderly, for example, and perhaps no longer very mobile, it is often the case that relatives accompany them to appointments, drive them there, help them fill out documents and submit them, and find this very stressful. (I24: 39)

Relatives and friends often provide emotional support and may be present at the time of death. Interviewees noted that, depending on the method used, the level of active involvement required from relatives differs. While no direct action is needed with the intravenous method, the oral method may require relatives to prepare and administer the medication.

Then [with the oral method], the suicide assistant discusses all the necessary steps with the relative, hands them the medication, and they do it. So then they [the individual who wishes to die and their relatives] have a bit more freedom in terms of timing. (I21: 79)

Interviewees also raised the issue that, following the death, relatives and friends may require support in processing the experience and dealing with grief. However, they were aware of only a few regular support services available for those who had accompanied someone through assisted suicide, whether offered by right-to-die organisations or other providers.

Well, I know that there is currently an initiative in place 3 to offer counselling for relatives of people who have committed suicide. But as far as I know, there is no regular service offered by right-to-die organisations, for example, specifically for relatives. (I11: 63)

### Funeral directors

According to interviewees, funeral directors are typically involved in assisted suicide only after the individual has died, when they assume responsibility for collecting and preparing the body for burial. However, in certain cases, funeral directors may become involved at an earlier stage. Some interviewees referenced funeral directors who provide information on their websites regarding the possibility of assisted suicide. Others referred to funeral directors who offer practical support, such as arranging for physicians to provide suicide assistance on a commercial basis or making their premises available for the procedure.

There is this funeral director who makes his work public. So in a way, he is also doing a bit of advertising, because you can go to his website and find him. The other one is involved in this field because of her long-standing contact with suicide assistant A, which is how she gets the cases, or rather how suicide assistant A comes to her, and I think her role is to provide a space and collaborate with a physician on a regular basis. (I9: 140–141)

However, as one interviewee, who had not been involved directly in assisted suicide, noted, some funeral directors only make their premises available for assisted suicide on an ad hoc basis, such as when no other location is available.

In this case, there were no premises available for assisted suicide. And then the funeral director in the village A agreed to make premises available in her funeral home. (I11: 68)

Some interviewees described assisted suicide as having become an extended service offering of certain funeral directors. In particular, one right-to-die organisation, originally founded as a funeral home and currently operated by a former funeral director, was mentioned.

There are now funeral homes that have discovered this as a gap in the market and also offer this service: ‘If you do it with us, you can do it on our premises and we will then practically send you straight to the coffin.’ I’m exaggerating, but this is now also a business model. (I25: 67)

### Indirectly involved players

In addition to those directly involved in assisted suicide, a number of other professional groups may be involved on an ad hoc or regular basis. Indirectly involved players do not generally support assisted suicides but may in some cases and under certain circumstances be involved in the process. As outlined in the interviews, these groups and their associated tasks include: pharmacists (responsible for the provision of medication), lawyers and psychologists (involved in assessing free will within the context of right-to-die organisations), emergency services and emergency doctors (responsible for confirming death, conducting post-mortem examinations and managing any complications that arise during the suicide), the criminal investigation department of the police (tasked with securing the scene and collecting evidence), and public prosecutors (who investigate unnatural deaths). Another topic raised in the interviews was the potential involvement of nurses working in in-patient or outpatient geriatric care. While not directly involved in the act of assisted suicide, nurses may witness the procedure and support relatives and friends before and after the event.

Nurses were not directly involved in the actual practice of suicide assistance, of course, but they were there to provide mental support, to help prepare for the last day and to fulfil the last wishes. They also provided emotional support and someone to talk to. (I12: 59)

Interviewees were uncertain about the extent to which outpatient hospice services and other end-of-life care providers are involved in assisted suicide.

## Discussion

The present work offers an overview of the providers of suicide assistance and other participants in this process in Germany, based on qualitative interviews with national experts on suicide assistance. The findings show that right-to-die organisations play a pivotal role in the practice of assisted suicide, providing information and counselling, assessing the legitimacy of the wish to die, organising assisted suicides and often actively contributing to their implementation. Both medical and non-medical suicide assistants routinely offer assistance to a heterogeneous group of individuals, both within and beyond the scope of right-to-die organisations. Treating physicians, relatives and even funeral directors may occasionally facilitate assisted suicide, while numerous other players appear to be indirectly involved.

### Heterogeneity of providers in an unregulated field

Compared to all other countries, Germany has the fewest legal restrictions on assisted suicide. There are no limitations on who may offer suicide assistance, no guidelines governing the procedure or means employed, and no restrictions on its recipients [1]. In contrast to the regulatory frameworks found in other countries – where access to assisted suicide is either state-regulated (e.g., Austria [37]) or administered by healthcare professionals (e.g., Canada [38]) – providers of suicide assistance in Germany are not subject to any form of regulation. The present results indicate that, in addition to right-to-die organisations and physician-assisted providers, an increasing number of professionals from diverse backgrounds are contributing to suicide assistance. While right-to-die organisations adhere to certain self-developed standards – which are made transparent to varying degrees – the activities of non-affiliated suicide assistants are largely unknown and opaque. While a local study from the Greater Munich area found that assisted suicide predominantly takes place within the context of right-to-die organisations [27], it is likely that a significant number of cases go unreported.

Physicians appear to play a pivotal role in the provision of suicide assistance, both within and outside right-to-die organisations [27]. They are often the first point of contact for individuals contemplating suicide and are responsible for assessing the individual’s suicidal intent. In addition, they organise medication, administer infusion therapy and conduct the post-mortem examination. This accumulation of responsibilities is considered problematic within right-to-die organisations, due to the absence of review by a neutral party. Outside these organisations, it is likely to be even more problematic, as demonstrated by initial court rulings on the matter [39]. The present findings further suggest that, in addition to right-to-die organisations and medical practitioners, other professional groups (e.g., nurses, funeral directors) also participate in assisted suicide, though their precise activities remain largely obscure. The findings of our study on assisted suicide outside the context of right-to-die organisations, as well as the scope of practice of non-medical suicide assistants, are based primarily on the accounts of individuals who were not directly involved in assisted suicide. The absence of precision in the findings necessitates further qualitative and quantitative research.

### Lack of independent information and consultation

In Germany, there is no national record of assisted suicides, unlike in Austria [37] and Canada [38]. Furthermore, specific legal requirements, professional guidelines and impartial information regarding the provision of suicide assistance are lacking. As a result, both individuals contemplating suicide and healthcare professionals face considerable uncertainty on this matter [31,40]], and predominantly turn to right-to-die organisations for information. These organisations have developed practices that are largely compliant with existing legal frameworks, and they frequently invite physicians to participate, thereby acting as central multipliers of knowledge. However, the multifaceted roles assumed by right-to-die organisations and certain suicide assistants – encompassing information provision, consultation, organisation and implementation – give rise to potential conflicts of interest. The question of how independent and neutral such organisations and individuals can be when they stand to benefit financially from the use of assisted suicide remains unresolved. While some general practitioners and palliative care physicians offer counselling and assistance in individual cases, there is no autonomous and impartial body providing information and guidance on assisted suicide at a general level. A valuable area for future research is thus the integration of information about death wishes and assisted suicide into the training and continuing education of healthcare professionals, as has been successfully implemented in Canada [14] and Austria [37].

### The (invisible) involvement of relatives and friends

On average, each death affects five to nine relatives, who often take on caregiving roles and experience grief in the aftermath [41,42]. In the specific context of assisted suicide, the individual’s active wish to die adds complexity to the roles and experiences of relatives and friends [43,44]. On the one hand, they may act as supportive figures and caregivers; while on the other hand, they may struggle with the decision and attempt to dissuade their loved one from proceeding. The present results suggest that relatives and friends are sometimes actively involved in the organisation of assisted suicide in Germany. As in Switzerland [45] and Belgium [25], they may assist the individual contemplating suicide by gathering information, contacting right-to-die organisations, completing documentation and coordinating the process. Right-to-die organisations and suicide assistants generally seek to involve relatives in the process. They may also be present during the dying process. In the rare cases where assisted suicide is carried out via oral administration of medication [46], relatives and friends may even be responsible for providing the medication. Although research on assisted suicide has typically viewed relatives as mourners, the present findings underscore their active involvement in the preparation and organisation of such deaths. However, it remains unclear what kind of support they require after assisting in a suicide, and to what extent such support services are available in the German context.

### Strengths and limitations

A key strength of the present study lies in its inclusion of experts representing a range of perspectives and roles, while possessing both theoretical and practical knowledge of assisted suicide in Germany. The data provided in the interviews reflect providers’ and participants’ broad expertise, including insights into informal practices involving independent and non-physician suicide assistants, funeral directors and relatives.

Of note, fewer than half of the interviewees had direct experience of suicide assistance and were able to provide first-hand knowledge. The remaining interviewees had derived their understanding from personal research and engagement in specialist discourse. This indirect knowledge must be interpreted with caution and verified through empirical research. Caution is also warranted when interpreting interviewees’ assessments of the frequency with which particular providers and participants are involved in assisted suicide, as no concrete data exist to substantiate these estimates. The under-representation of interviewees who were directly involved in assisted suicide may also indicate that informal practices in assisted suicide are rarely reported.

## Conclusions

The present work provides insight into the current practice of assisted suicide in Germany by highlighting the perspectives of its providers and participants. While right-to-die organisations and individual suicide assistants offer information and services based on standardised procedures, comparatively little is known about the roles of treating physicians, funeral directors, relatives and other indirectly involved players (e.g., nurses, police, hospice staff). Given the minimal regulation of assisted suicide in Germany, the landscape of providers and supporters appears to be diversifying and growing increasingly complex. These results appear to be linked to the current regulation of assisted suicide in Germany. Consequently, the application of this model to other countries is not feasible.

Future research should therefore examine the roles, experiences, challenges and best practices of the various actors involved in the provision of assisted suicide.

## Supporting information

S1 File Interview guide. (DOCX)

S2 File Sociodemographic questionnaire. (DOCX)

S3 File COREQ-Checklist. (DOCX)
