# Supplementary material for: Providers of and participants in suicide assistance in Germany: A field description based on expert interviews
Source: PLoS One. 2026 Jun 18;21(6):e0350926. doi: 10.1371/journal.pone.0350926 (PMC13278400; doi:10.1371/journal.pone.0350926)
Supplement: S1 File — (DOCX) [file pone.0350926.s001.docx]

**Appendix 1: Interview guide**

| **Experiences of interviewees regarding assisted suicide** |
| --- |
| In our study, we are interested in how assisted suicide is currently being practised in Germany. We have selected you as an interview partner due to your expertise on the subject.  Could you begin by telling me where you acquired your knowledge and experience of assisted suicide? |
| **Open introductory questions** |
| I would now like to ask you a few questions about how assisted suicide is currently being practised in Germany:   - What options are available to individuals seeking assisted suicide? - To your knowledge, how is assisted suicide practised in Germany? - Where does assisted suicide take place in Germany? - What do you know about the individuals who seek assisted suicide?   - Motives?   - Life situations?   - Characteristics? |
| **Information and advice** |
| Next, I would like to discuss the areas of action and topics involved in assisted suicide, beginning with information and counselling options:   - How do individuals obtain information about assisted suicide? - Who provides counselling for individuals seeking assisted suicide? - How do healthcare professionals obtain information on assisted suicide? - What options are available to healthcare professionals seeking advice on assisted suicide? |
| **Preparation and organisation** |
| How is it determined whether the decision to pursue assisted suicide is freely made and permanent?   - Who carries out this assessment? - What criteria are used to make this assessment? - On what grounds is assisted suicide refused?   - How do individuals cope when their request for assisted suicide is refused? - How is it ensured that the procedure is carried out correctly and safely?   - Who is responsible for ensuring this? - How do individuals obtain the necessary medication? - To what extent are friends and relatives involved? - What are the costs of assisted suicide?   - What specific costs are involved? |
| **Performance of assisted suicide** |
| - What medications are used?   - How are these medications administered? - To what extent is the performance of assisted suicide supervised? - What happens if complications arise? - Who provides care afterwards? - Which individuals are present during the procedure? - Who else could be present? |
| **After death** |
| What happens after death?   - How is death determined? - What happens to the body? - How are the police informed? - How are relatives and friends informed? - To what extent are relatives and friends supported afterwards? |
| **Individuals and organisations involved** |
| We have already discussed the various individuals and actors involved in assisted suicide. I would now like to ask a more specific question: To your knowledge, which individuals are involved in assisted suicide, and what roles do they play? For example:   - Physicians (e.g., general practitioners, palliative care specialists, etc.)? - Nursing staff? - Psychotherapists? - Pharmacists? - Funeral directors? - Lawyers and solicitors? - Euthanasia associations? - Hospice services? - Relatives and friends?   Which other professional groups are involved in assisted suicide?  Which other organisations play a role in assisted suicide? |
| **Challenges** |
| In your opinion, what problems exist in the practice of assisted suicide in Germany?   - What challenges are faced by:   - Individuals seeking assisted suicide?   - Relatives and friends?   - Healthcare professionals? - To what extent do you see a need for legal or organisational regulation of assisted suicide? - What would help to make assisted suicide safer for everyone involved in Germany? |
| **Outlook** |
| - In your opinion, are there any other issues that are important for the practice of assisted suicide? - Are there any other matters of importance to you? |

**Thank you for participating in this interview**
