# Supplementary material for: Providers of and participants in suicide assistance in Germany: A field description based on expert interviews
Source: PLoS One. 2026 Jun 18;21(6):e0350926. doi: 10.1371/journal.pone.0350926 (PMC13278400; doi:10.1371/journal.pone.0350926)
Supplement: S2 File — (DOCX) [file pone.0350926.s002.docx]

**Appendix 2: sociodemographic questionnaire**

| **1. Sex:** |  | |  | | | | |  |
| --- | --- | --- | --- | --- | --- | --- | --- | --- |
| .  male | female | | .  diverse | | | | |  |
| **2. Age:** _______ years | | | | | | | |  |
| **3. In which federal state are you currently living?** | | | | | | | |  |
| ☐ Baden-Württemberg  ☐ Bavaria  ☐ Berlin  ☐ Brandenburg | | ☐ Bremen  ☐ Hamburg  ☐ Hesse  ☐ Mecklenburg-Western Pomerania | | | | ☐ Lower Saxony  ☐ North Rhine-Westphalia  ☐ Rhineland-Palatinate  ☐ Saarland | ☐ Saxony-Anhalt  ☐ Saxony  ☐ Schleswig-Holstein  ☐ Thüringia |  |
| **4. What is your highest level of education?** | | | | | | | |  |
| Apprenticeship or technical college education  Technical college (master craftsman/business administrator/technician)  Bachelor's degree or intermediate diploma  Master's degree or diploma/examination/magister | | | | | Doctorate  I am still in training/studying  Other training (e.g. acquired abroad): ________________  No vocational qualification and not in training/studying | | |  |
| **5. In which field are you currently working?** Multiple answers possible. | | | | | | | |  |
| .  Research/science  .  Journalism  .  (Expert) Association | | | | .  Patient care  .  Other, namely: ________________ | | | |  |
| **6. Job title: __________________________________** | | | | | | | |  |
| **7. What is your professional or educational background?** Multiple answers possible | | | | | | | |  |
| . ☐ Medicine  . ☐ Nursing/nursing sciences.  . ☐ Law/legal studies  ☐ Journalism | | | | ☐ Humanities/social sciences/public health  ☐ Philosophy/ethics  ☐ Psychology  ☐ Other, namely: ________________ | | | |  |
| **8. How many years have you been involved with the issue of assisted suicide?** Approximately _____ years. | | | | | | | |  |
| **9. Have you ever been involved in assisted suicides?** | | | | | | | |  |
| . ☐ Yes ☐ No. ☐ not specified | | | | | | | |  |
