# Supplementary material for: Providers of and participants in suicide assistance in Germany: A field description based on expert interviews
Source: PLoS One. 2026 Jun 18;21(6):e0350926. doi: 10.1371/journal.pone.0350926 (PMC13278400; doi:10.1371/journal.pone.0350926)
Supplement: Attachment — Submitted filename: Response to Reviewers.docx [file pone.0350926.s005.docx]

PLOS ONE

| 1. Please ensure that your manuscript meets PLOS ONE's style requirements, including those for file naming. The PLOS ONE style templates can be found at… | The manuscript now meets PLOS ONE’s style requirements, including those for file naming. |
| --- | --- |
| financial disclosure statement  Please state what role the funders took in the study. If the funders had no role, please state: "The funders had no role in study design, data collection and analysis, decision to publish, or preparation of the manuscript." | The statement has been changed as requested. |
| Data availability  All PLOS journals now require all data underlying the findings described in their manuscript to be freely available to other researchers, either a. In a public repository, b. Within the manuscript itself, or c. Uploaded as supplementary information. This policy applies to all data except where public deposition would breach compliance with the protocol approved by your research ethics board. If your data cannot be made publicly available for ethical or legal reasons (e.g., public availability would compromise patient privacy), please explain your reasons on resubmission and your exemption request will be escalated for approval. | The raw data related to this publication cannot be openly released, as some restrictions due data protection regulations apply. The data contain transcripts of interviews and no interviewees consented to having the complete transcription of their interview shared.  This limitation has been added in the Data Availability section. |
| If the reviewer comments include a recommendation to cite specific previously published works, please review and evaluate these publications to determine whether they are relevant and should be cited. There is no requirement to cite these works unless the editor has indicated otherwise. | We have evaluated the recommendation from reviewers to cite specific, previously published works that are relevant to our own. |

Review #1

| In my view, the framing and international relevance of the manuscript would benefit from the inclusion—either in the Introduction or in a brief contextual paragraph in the Discussion—of a concise overview of the most recent developments in Europe regarding the legalisation/regulation of assisted suicide (and, where relevant, euthanasia). Even a short summary indicating which countries have introduced regulatory changes in recent years and what key regulatory models are emerging would help readers better situate the German case. In this respect, comparative references such as: <https://pubmed.ncbi.nlm.nih.gov/32437262/> (10.1177/0025817220923687), <https://pubmed.ncbi.nlm.nih.gov/34330794/> (10.1136/medethics-2021-107523) could be helpful. | Thank you very much for your valuable comment on the placement of the German legal situation within the European context, and for the references you provided. The introduction has been updated to include a corresponding explanation, as well as a reference to the legal frameworks surrounding assisted suicide in European countries.  “This trend in Germany towards the liberalisation of assisted dying is in line with developments in other European countries in recent years. Following the long-standing legalisation of assisted suicide in Switzerland and of assisted suicide and euthanasia in the Netherlands and Belgium, Italy (2019), Austria (2020), Spain (2021), and France (2025) have also introduced legislation regulating assisted suicide and, in some cases, euthanasia since 2019.” |
| --- | --- |
| A second issue concerns the composition of the sample: only 5 of the 16 interviewees report direct involvement in suicide assistance, whereas the others primarily provide indirect knowledge (through research, observation of the public debate, or related professional roles). Although this limitation is already acknowledged by the authors, I believe it should be handled in a more structured way because it affects the strength of the inferences: describing “how the practice works” is not equivalent to describing “how it is reconstructed/perceived by informed stakeholders.” I would therefore recommend distinguishing more systematically—both in the analysis and in the presentation of findings—between informants with direct versus indirect experience, and clarifying how this difference informed coding, interpretation, and the weight attributed to different strands of evidence. | Thank you for this important comment.  All statements in the Results section were reviewed once again to verify who had made them. Any statements made solely by individuals who had not yet been directly involved in assisted suicide were introduced accordingly. This procedure is explained at the beginning of the presentation of the interview findings: “Findings derived exclusively from individuals who had not yet been directly involved in assisted suicide are expressly mentioned.”  In the Discussion section, the absence of experts who had been directly involved in assisted suicide was highlighted in relation to the topics ‘assisted suicide outside of right-to-die organisations’ and ‘non-medical assisted suicide providers’:  “The findings of our study on assisted suicide outside of right-to-die organisations, as well as the scope of practice of non-medical suicide assistants, are based primarily on the accounts of individuals who were not directly involved in assisted suicide. The absence of precision in the findings necessitates further research.” |
| Finally, while I appreciate the mapping of actors, the Results section currently risks reading primarily as a taxonomy of roles and tasks (as summarised in the code tree) rather than a full field description. The categories identified are plausible (organisations, assistants, treating physicians, funeral services, family members, and indirect actors), but it remains less clear how the field operates in practice: what the typical pathways look like, the temporal sequence in which different actors become involved, the minimum steps in the process (from request to death and subsequent procedures), and where the main variants or decision points lie (for instance, differences between organisation-mediated pathways and non-affiliated routes, or the possible involvement of institutional actors). Even a brief, schematic representation of pathways (e.g., as a box or figure) would substantially enhance clarity and added value. | Thank you very much for this interesting suggestion. Unfortunately, it is not possible to provide a detailed description of individual pathways based on the expert interviews, but this will be done in the next phase of the research project.  A schematic overview of the assisted suicide process has now been included. |
| In this context, the distinction between “providers” and “participants” could also be made more consistent throughout the text and the code tree by more clearly specifying the operational criteria and the positioning of indirect actors; this would help to better substantiate certain statements in the Discussion (e.g., “increasing complexity/heterogeneity”), which currently appear more asserted than demonstrated in a structured way. | In the code tree, the term “indirect involved providers and participants” was replaced by the term “indirectly involved players” as it is used in the Results section.  In the Results section, the term “indirectly involved players” is concretised:  “Indirectly involved players do not generally support assisted suicides, but may in some cases and under certain circumstances be involved in the process.”  In the Discussion Section, the term “indirectly involved players” was used consistently, and a passage that could have been misinterpreted was clarified:  “Treating physicians, relatives and even funeral directors may occasionally facilitate assisted suicide, while numerous other players appear to be indirectly involved.” |
| Given the sensitivity of the topic, I also recommend consistent terminology (e.g., using “right-to-die organisations” uniformly), avoiding non-equivalent terms (such as “euthanasia organisations”), and clearly defining the key terms adopted. | The term ‘euthanasia organisations’ has been consistently replaced by the term ‘right-to-die organisations’. |

Review #2

| My main concern is the Data Availability Statement. As written, the statement that data are available from the corresponding author upon reasonable request does not appear sufficient for PLOS ONE. If full public sharing is not possible because of ethical or legal concerns, the manuscript should clearly explain those restrictions and provide a journal-compliant restricted-access pathway. | The raw data related to this publication cannot be openly released, as some restrictions due data protection regulations apply. The data contain transcripts of interviews and no interviewees consented to having the complete transcription of their interview shared.  This limitation has been added in the Data Availability section. |
| --- | --- |
| Second, the analytic framing should be more precise. The manuscript is strongest as a structured expert-based field description. Because the interview guide was substantially literature-informed and included prompts about specific actors, the findings should not be presented as if the actor categories emerged purely inductively. The authors should frame the paper more explicitly as a qualitative field-mapping study. | Thank you very much for this important point. The coding process relating to the providers and participants has been clarified in the Methods section.  “Some categories of providers and participants were derived deductively from the literature (e.g. right-to-die organisations, physicians, relatives and friends, nurses), whilst others were developed inductively from the data. In some cases, deductive categories were refined on the basis of the data (e.g. treating physicians and physician suicide assistants).” |
| Third, several conclusions should be stated more cautiously. Fewer than half of participants had direct involvement in assisted suicide, and much of the evidence reflects expert knowledge rather than direct observation. Accordingly, claims about practice in Germany should more often be phrased as what interviewees described or what the findings suggest. | Thank you for this important comment.  All statements in the Results section were reviewed once again to verify who had made them. Any statements made solely by individuals who had not yet been directly involved in assisted suicide were introduced accordingly.  This procedure is explained at the beginning of the presentation of the interview findings:  “Findings derived exclusively from individuals who had not yet been directly involved in assisted suicide are expressly mentioned.”  In the Discussion section, the absence of experts who had been directly involved in assisted suicide was highlighted in relation to the topics ‘assisted suicide outside of right-to-die organisations’ and ‘non-medical assisted suicide providers’.  “The findings of our study on assisted suicide outside of right-to-die organisations, as well as the scope of practice of non-medical suicide assistants, are based primarily on the accounts of individuals who were not directly involved in assisted suicide. The absence of precision in the findings necessitates further research.” |
| The Methods section would also benefit from fuller reporting of qualitative rigor, especially sample adequacy, coding review, handling of disagreements, and reflexivity. | The Methods section was supplemented:  Sampling  “The interview study was concluded once all identified interviewees had been interviewed and no further interviews revealed any additional providers, participants or interviewees.”  Data analysis  “Some categories of providers and participants were derived deductively from the literature (e.g. right-to-die organisations, physicians, relatives and friends, nurses), whilst others were developed inductively from the data. In some cases, deductive categories were refined on the basis of the data (e.g. treating physicians and physician suicide assistants).” |
| In addition, terminology should be standardized throughout, | The term ‘euthanasia organisations’ has been consistently replaced by the term ‘right-to-die organisations’. |
| and the limitations section should more clearly acknowledge likely underrepresentation of less visible or informal practices. | The Limitations section has been supplemented:  “The under-representation of interviewees who were directly involved in assisted suicide may also indicate that informal practices in assisted suicide are rarely reported.” |

Review #3

| I would suggest that the abstract in question would benefit from a better form of framing of the context of the study. This would allow a better understanding of the importance of the topic. Your conclusions should also be improved. | Due to the limited character count in the abstract, the Background section could not be expanded. However, in the introduction of the main manuscript, the German context is now more firmly situated within the broader European context. |
| --- | --- |
| Keywords: are these keywords Mesh terms? | Mesh terms were revised and the term “Death, ~~Assisted,~~” was corrected. |
| As far as I can see, the literature addressed has not been described accurately. The overall organization and clarity throughout the manuscript should be improved. For the introduction, a restructuring of the writing to provide more coherent and connected ideas and sections would be valuable. A brief synopsis or synthesis of ideas and relationships between or within constructs would improve flow dramatically. It is not clear what knowledge this paper adds from an international perspective. How is this/your study increasing our understanding/knowledge? | Thank you very much for your valuable comment regarding the placement of the German legal situation within the European context. A corresponding explanation has been included in the introduction.  “This trend in Germany towards the liberalisation of assisted dying is in line with developments in other European countries in recent years. Following the long-standing legalisation of assisted suicide in Switzerland and of assisted suicide and euthanasia in the Netherlands and Belgium, Italy (2019), Austria (2020), Spain (2021), and France (2025) have also introduced legislation regulating assisted suicide and, in some cases, euthanasia since 2019.” |
| Please provide the specific questions under study, as it would be helpful for the readers. | The research question has been added in the study aim section:  The research question is: Which providers and participants of assisted suicides in Germany exist and which tasks do they perform? |
| The ethical and consent elements should be moved to the end of the Methods section. | The ethical and consent elements have been moved to the end of the Methods section. |
| More information about the study approach, along with its epistemological underpinnings, is, therefore, needed. This needs to be expanded, clarified, and supported by in-text citations. Authors need to pay close attention to the requirements of reporting guidelines for qualitative research (e.g., COREQ checklist). | In the design section it was added, that the study followed an interpretative approach.  “The ASEP-study was a prospective, observational qualitative investigation that explored and analysed the practice of assisted suicide in Germany following an interpretative approach (33).”  Missing steps of the COREQ guideline have been added (Items 6-8, 23, 28). |
| The authors do not follow all the steps of COREQ, such as the presentation of the characteristics of the research context, what kind of relationship the researcher has with the participants, and how the participants were recruited. | Missing steps of the COREQ guideline have been added (Items 6-8, 23, 28). |
| Interviews: Interview guide was it pilot-tested? Were repeat interviews carried out? | As described in the Methods section two pilot interviews were conducted.  As no repeat interviews were conducted, this was not reported. |
| What strategies were developed in the interviews to create rapport with the participants and deal with communication impasses? | Information was added in the Data Collection section:  “The interviews commenced with an introductory question. This enabled the participants to introduce themselves and establish a rapport with the interviewer. If an interviewee did not understand a question, it was repeated using different words. If an interviewee did not wish to answer a question, it was omitted.” |
| Were transcripts returned to participants for comment and/or correction? | Information was added at the end of the Data Collection section:  “None of the participants requested to have their transcripts returned. Participants were not asked to comment on the results of the study.” |
| It is unclear how the authors will ensure the reliability of qualitative coding. Provide this information. | We state in the Data Analysis section, the reliability of the qualitative coding was reviewed by a second member of the research team. |
| The process of analysis should be made as transparent as possible (notwithstanding the conceptual and theoretical creativity that typically characterizes qualitative research). Did participants provide feedback on the findings (triangulation)? How do you ensure methodological rigour? The researcher’s own position should clearly be stated. For example, have they examined their own role, possible bias, and influence on the research (reflexivity)? What experience or training did the researchers have? Did they use reflexive bracketing to achieve openness? | The process of data collection and data analysis has been concretised:  Data collection  “None of the participants requested to have their transcripts returned. Participants were not asked to comment on the results of the study.”  Data analysis  “Some categories of providers and participants were derived deductively from the literature (e.g. right-to-die organisations, physicians, relatives and friends, nurses), whilst others were developed inductively from the data. In some cases, deductive categories were refined on the basis of the data (e.g. treating physicians and physician suicide assistants).”  Moreover, the background of the researcher was added in the Data Collection section:  “The interviewer had no personal, commercial or political interest in the research topic.”  “All interviews were conducted by the first author, who is a trained sociologist with a rigorous training in qualitative and quantitative methods of empirical social research.” |
| In the data collection, the verification of transcript completeness and accuracy relied on cross-checking among researchers and somewhat employing triangulation methods. Clearer documentation of the coding process and how data saturation was determined would enhance the credibility of the findings. | In the Sampling section it was added:  “The interview study was concluded once all identified interviewees had been interviewed and no further interviews revealed any additional providers, participants or interviewees.” |
| Findings were not explicitly interrelated; there was no presentation of a structure. I suggest an illustration/diagram for a better analysis. | A schematic overview of the assisted suicide process is included. |
| The findings are interesting; however, more samples of data would be welcome. Additionally, while quantitative detail is not the focus, it may be useful to provide some indication of whether these experiences were shared by the majority of participants or by only a few. | Where appropriate, quantitative details regarding the interviewees were provided. Furthermore, explicit mention was made where statements were made only by individuals who were not directly involved in assisted suicide.  “Interviewees who had not themselves been involved directly in assisted suicide also stated that….” |
| There are some conclusions drawn that have neither a literature review nor research to support them. It is also not entirely clear whether deductive or inductive logic is employed here. | Unfortunately, we were unable to verify this comment. |
| In the discussion section, the recommendations for practice/research/education should have been approached in greater depth. Please discuss whether or how the findings can be transferred to other populations, or consider other ways the research may be used. | As the findings primarily highlight gaps in the research and are based solely on the assessments of experts who were only partially directly involved in assisted suicides, we should exercise caution when drawing specific implication for practice and avoid over-interpreting the results.  It was added, that further qualitative and quantitative research is needed:  “The findings of our study on assisted suicide outside the context of right-to-die organisations, as well as the scope of practice of non-medical suicide assistants, are based primarily on the accounts of individuals who were not directly involved in assisted suicide. The absence of precision in the findings necessitates further qualitative and quantitative research.” |
| Theoretical and methodological limitations should be deeply emphasised. Please also explore the strengths of this study that make it unique and distinguish it from other qualitative studies. | Strengths and limitations are mentioned in the Limitations section. The limitation regarding the under-representation of interviewees, who were directly involved in assisted suicides, was concretised:  “The under-representation of interviewees who were directly involved in assisted suicide may also indicate that informal practices in assisted suicide are rarely reported.” |
| Give the reader some deeper insight in your conclusion section. Still, broad generalisations should be avoided, and the regional specificity of the study context should be reinforced. Checklist for style | Thank you for this important comment. We now give deeper insights in our Conclusion:  “These results appear to be linked to the current regulation of assisted suicide in Germany. Consequently, the application of this model to other countries is not feasible.” |
| The manuscript needs to be carefully and attentively proofread. I would recommend a thorough technical edit of this paper. | The entire manuscript has been professionally proofread by a native English speaker whom we acknowledge in our Acknowledgements section. |
