# Supplementary material for: Providers of and participants in suicide assistance in Germany: A field description based on expert interviews
Source: PLoS One. 2026 Jun 18;21(6):e0350926. doi: 10.1371/journal.pone.0350926 (PMC13278400; doi:10.1371/journal.pone.0350926)
Supplement: Peer review history

# Peer review: Decision Letter 0

**DOI:** 10.1371/journal.pone.0350926.r001

**Authors:** Andrea Cioffi

26 Mar 2026

PONE-D-25-56777

Providers of and participants in suicide assistance in Germany: A field description based on expert interviews

PLOS One

Dear Dr. Schwabe,

Thank you for submitting your manuscript to PLOS ONE. After careful consideration, we feel that it has merit but does not fully meet PLOS ONE’s publication criteria as it currently stands. Therefore, we invite you to submit a revised version of the manuscript that addresses the points raised during the review process.

We note that some of the comments of the reviewers refer to specific articles for you to cite. Please note that it is not mandatory that you cite these specific articles and you are welcome to seek alternatives manuscripts in the literature that are relevant to your manuscript’s content.

Please submit your revised manuscript by May 10 2026 11:59PM. If you will need more time than this to complete your revisions, please reply to this message or contact the journal office at plosone@plos.org. When you're ready to submit your revision, log on to https://www.editorialmanager.com/pone/ and select the 'Submissions Needing Revision' folder to locate your manuscript file.

Please include the following items when submitting your revised manuscript:

- A letter that responds to each point raised by the academic editor and reviewer(s). You should upload this letter as a separate file labeled 'Response to Reviewers'.
- A marked-up copy of your manuscript that highlights changes made to the original version. You should upload this as a separate file labeled 'Revised Manuscript with Track Changes'.
- An unmarked version of your revised paper without tracked changes. You should upload this as a separate file labeled 'Manuscript'.

If you would like to make changes to your financial disclosure, please include your updated statement in your cover letter. Guidelines for resubmitting your figure files are available below the reviewer comments at the end of this letter.

If applicable, we recommend that you deposit your laboratory protocols in protocols.io to enhance the reproducibility of your results. Protocols.io assigns your protocol its own identifier (DOI) so that it can be cited independently in the future. For instructions see: https://journals.plos.org/plosone/s/submission-guidelines#loc-laboratory-protocols. Additionally, PLOS ONE offers an option for publishing peer-reviewed Lab Protocol articles, which describe protocols hosted on protocols.io. Read more information on sharing protocols at https://plos.org/protocols?utm_medium=editorial-email&utm_source=authorletters&utm_campaign=protocols.

We look forward to receiving your revised manuscript.

Kind regards,

Andrea Cioffi

Academic Editor

PLOS One

Journal Requirements:

When submitting your revision, we need you to address these additional requirements.

1. Please ensure that your manuscript meets PLOS ONE's style requirements, including those for file naming. The PLOS ONE style templates can be found at

https://journals.plos.org/plosone/s/file?id=wjVg/PLOSOne_formatting_sample_main_body.pdf and

https://journals.plos.org/plosone/s/file?id=ba62/PLOSOne_formatting_sample_title_authors_affiliations.pdf

2. Thank you for stating the following financial disclosure:

The study “ASEP – Assisted suicide in Germany: Exploring the practice” is funded (on the basis of peer reviews) by the German Research Foundation (DFG) (Project N° 537566039). The grant was awarded to SvS. The funding body was not involved in the study design, the preparation of this paper, or the decision to submit the paper for publication. The funding body will not be involved in the collection, analysis, or interpretation of the study data.

Please state what role the funders took in the study. If the funders had no role, please state: "The funders had no role in study design, data collection and analysis, decision to publish, or preparation of the manuscript."

If this statement is not correct you must amend it as needed.

Please include this amended Role of Funder statement in your cover letter; we will change the online submission form on your behalf.

3. In the online submission form, you indicated that the datasets generated and/or analysed during the current study are available from the corresponding author upon reasonable request. All relevant data will be made available upon study completion.

All PLOS journals now require all data underlying the findings described in their manuscript to be freely available to other researchers, either a. In a public repository, b. Within the manuscript itself, or c. Uploaded as supplementary information.

This policy applies to all data except where public deposition would breach compliance with the protocol approved by your research ethics board. If your data cannot be made publicly available for ethical or legal reasons (e.g., public availability would compromise patient privacy), please explain your reasons on resubmission and your exemption request will be escalated for approval.

4. If the reviewer comments include a recommendation to cite specific previously published works, please review and evaluate these publications to determine whether they are relevant and should be cited. There is no requirement to cite these works unless the editor has indicated otherwise.

[Note: HTML markup is below. Please do not edit.]

Reviewers' comments:

Reviewer's Responses to Questions

**Comments to the Author**

1. Is the manuscript technically sound, and do the data support the conclusions?

The manuscript must describe a technically sound piece of scientific research with data that supports the conclusions. Experiments must have been conducted rigorously, with appropriate controls, replication, and sample sizes. The conclusions must be drawn appropriately based on the data presented.

Reviewer #1: Partly

Reviewer #2: Yes

Reviewer #3: Partly

**********

2. Has the statistical analysis been performed appropriately and rigorously?

Reviewer #1: I Don't Know

Reviewer #2: N/A

Reviewer #3: N/A

**********

3. Have the authors made all data underlying the findings in their manuscript fully available?

The PLOS Data policy requires authors to make all data underlying the findings described in their manuscript fully available without restriction, with rare exception (please refer to the Data Availability Statement in the manuscript PDF file). The data should be provided as part of the manuscript or its supporting information, or deposited to a public repository. For example, in addition to summary statistics, the data points behind means, medians and variance measures should be available. If there are restrictions on publicly sharing data—e.g. participant privacy or use of data from a third party—those must be specified.

Reviewer #1: Yes

Reviewer #2: No

Reviewer #3: Yes

**********

4. Is the manuscript presented in an intelligible fashion and written in standard English?

PLOS ONE does not copyedit accepted manuscripts, so the language in submitted articles must be clear, correct, and unambiguous. Any typographical or grammatical errors should be corrected at revision, so please note any specific errors here.

Reviewer #1: Yes

Reviewer #2: Yes

Reviewer #3: Yes

**********

5. Review Comments to the Author

Please use the space provided to explain your answers to the questions above. You may also include additional comments for the author, including concerns about dual publication, research ethics, or publication ethics. (Please upload your review as an attachment if it exceeds 20,000 characters)

Reviewer #1: First, I would like to thank the Editor for the opportunity to review this manuscript.

The paper addresses a timely and relevant topic, particularly in light of the lack of comprehensive regulation and the limited availability of official data in Germany, and offers a qualitative mapping of the “actors” involved in suicide assistance through 16 expert interviews. The output (five main categories, together with additional indirect actors) is overall clear and potentially useful as a field description. Nevertheless, to make the contribution more robust and fully publishable in its current form, I would suggest a few targeted improvements.

In my view, the framing and international relevance of the manuscript would benefit from the inclusion—either in the Introduction or in a brief contextual paragraph in the Discussion—of a concise overview of the most recent developments in Europe regarding the legalisation/regulation of assisted suicide (and, where relevant, euthanasia). Even a short summary indicating which countries have introduced regulatory changes in recent years and what key regulatory models are emerging would help readers better situate the German case. In this respect, comparative references such as

: https://pubmed.ncbi.nlm.nih.gov/32437262/ (10.1177/0025817220923687), https://pubmed.ncbi.nlm.nih.gov/34330794/ (10.1136/medethics-2021-107523) could be helpful.

A second issue concerns the composition of the sample: only 5 of the 16 interviewees report direct involvement in suicide assistance, whereas the others primarily provide indirect knowledge (through research, observation of the public debate, or related professional roles). Although this limitation is already acknowledged by the authors, I believe it should be handled in a more structured way because it affects the strength of the inferences: describing “how the practice works” is not equivalent to describing “how it is reconstructed/perceived by informed stakeholders.” I would therefore recommend distinguishing more systematically—both in the analysis and in the presentation of findings—between informants with direct versus indirect experience, and clarifying how this difference informed coding, interpretation, and the weight attributed to different strands of evidence.

Finally, while I appreciate the mapping of actors, the Results section currently risks reading primarily as a taxonomy of roles and tasks (as summarised in the code tree) rather than a full field description. The categories identified are plausible (organisations, assistants, treating physicians, funeral services, family members, and indirect actors), but it remains less clear how the field operates in practice: what the typical pathways look like, the temporal sequence in which different actors become involved, the minimum steps in the process (from request to death and subsequent procedures), and where the main variants or decision points lie (for instance, differences between organisation-mediated pathways and non-affiliated routes, or the possible involvement of institutional actors). Even a brief, schematic representation of pathways (e.g., as a box or figure) would substantially enhance clarity and added value.

In this context, the distinction between “providers” and “participants” could also be made more consistent throughout the text and the code tree by more clearly specifying the operational criteria and the positioning of indirect actors; this would help to better substantiate certain statements in the Discussion (e.g., “increasing complexity/heterogeneity”), which currently appear more asserted than demonstrated in a structured way.

Given the sensitivity of the topic, I also recommend consistent terminology (e.g., using “right-to-die organisations” uniformly), avoiding non-equivalent terms (such as “euthanasia organisations”), and clearly defining the key terms adopted.

Reviewer #2: This manuscript addresses a timely and important topic and provides a useful qualitative description of the actors involved in assisted suicide in Germany. Strengths include the relevance of the research question, the appropriateness of expert interviews for an under-documented field, and a clear overall structure. The interview guide also appears well aligned with the study aim.

I recommend major revision before publication.

My main concern is the Data Availability Statement. As written, the statement that data are available from the corresponding author upon reasonable request does not appear sufficient for PLOS ONE. If full public sharing is not possible because of ethical or legal concerns, the manuscript should clearly explain those restrictions and provide a journal-compliant restricted-access pathway.

Second, the analytic framing should be more precise. The manuscript is strongest as a structured expert-based field description. Because the interview guide was substantially literature-informed and included prompts about specific actors, the findings should not be presented as if the actor categories emerged purely inductively. The authors should frame the paper more explicitly as a qualitative field-mapping study.

Third, several conclusions should be stated more cautiously. Fewer than half of participants had direct involvement in assisted suicide, and much of the evidence reflects expert knowledge rather than direct observation. Accordingly, claims about practice in Germany should more often be phrased as what interviewees described or what the findings suggest.

The Methods section would also benefit from fuller reporting of qualitative rigor, especially sample adequacy, coding review, handling of disagreements, and reflexivity. In addition, terminology should be standardized throughout, and the limitations section should more clearly acknowledge likely underrepresentation of less visible or informal practices.

Overall, this is a worthwhile manuscript with clear potential. With major revision, particularly on data availability, analytic framing, rigor reporting, and claim calibration, it could make a useful contribution.

Reviewer #3: I appreciate the opportunity to review this manuscript and hope my comments assist in the revision process. The material is interesting, and the topic is relevant. However, some areas need clarification, as noted below:

- I would suggest that the abstract in question would benefit from a better form of framing of the context of the study. This would allow a better understanding of the importance of the topic. Your conclusions should also be improved.

- Keywords: are these keywords Mesh terms?

- As far as I can see, the literature addressed has not been described accurately. The overall organization and clarity throughout the manuscript should be improved. For the introduction, a restructuring of the writing to provide more coherent and connected ideas and sections would be valuable. A brief synopsis or synthesis of ideas and relationships between or within constructs would improve flow dramatically. It is not clear what knowledge this paper adds from an international perspective. How is this/your study increasing our understanding/knowledge?

- Please provide the specific questions under study, as it would be helpful for the readers.

Materials and Methods

- The ethical and consent elements should be moved to the end of the Methods section.

- More information about the study approach, along with its epistemological underpinnings, is, therefore, needed. This needs to be expanded, clarified, and supported by in-text citations. Authors need to pay close attention to the requirements of reporting guidelines for qualitative research (e.g., COREQ checklist).

- The authors do not follow all the steps of COREQ, such as the presentation of the characteristics of the research context, what kind of relationship the researcher has with the participants, and how the participants were recruited.

- Interviews: Interview guide was it pilot-tested? Were repeat interviews carried out?

- What strategies were developed in the interviews to create rapport with the participants and deal with communication impasses?

- Were transcripts returned to participants for comment and/or correction?

- It is unclear how the authors will ensure the reliability of qualitative coding. Provide this information.

- The process of analysis should be made as transparent as possible (notwithstanding the conceptual and theoretical creativity that typically characterizes qualitative research). Did participants provide feedback on the findings (triangulation)? How do you ensure methodological rigour? The researcher’s own position should clearly be stated. For example, have they examined their own role, possible bias, and influence on the research (reflexivity)? What experience or training did the researchers have? Did they use reflexive bracketing to achieve openness?

- In the data collection, the verification of transcript completeness and accuracy relied on cross-checking among researchers and somewhat employing triangulation methods. Clearer documentation of the coding process and how data saturation was determined would enhance the credibility of the findings.

Findings

- Findings were not explicitly interrelated; there was no presentation of a structure. I suggest an illustration/diagram for a better analysis.

- The findings are interesting; however, more samples of data would be welcome. Additionally, while quantitative detail is not the focus, it may be useful to provide some indication of whether these experiences were shared by the majority of participants or by only a few.

Discussion

- There are some conclusions drawn that have neither a literature review nor research to support them. It is also not entirely clear whether deductive or inductive logic is employed here.

- In the discussion section, the recommendations for practice/research/education should have been approached in greater depth. Please discuss whether or how the findings can be transferred to other populations, or consider other ways the research may be used.

- Theoretical and methodological limitations should be deeply emphasised. Please also explore the strengths of this study that make it unique and distinguish it from other qualitative studies.

- Give the reader some deeper insight in your conclusion section. Still, broad generalisations should be avoided, and the regional specificity of the study context should be reinforced.

Checklist for style

- The manuscript needs to be carefully and attentively proofread. I would recommend a thorough technical edit of this paper.

**********

6. PLOS authors have the option to publish the peer review history of their article (what does this mean?). If published, this will include your full peer review and any attached files.

If you choose “no”, your identity will remain anonymous but your review may still be made public.

**Do you want your identity to be public for this peer review?**  For information about this choice, including consent withdrawal, please see our Privacy Policy.

Reviewer #1: No

Reviewer #2: No

Reviewer #3: No

**********

[NOTE: If reviewer comments were submitted as an attachment file, they will be attached to this email and accessible via the submission site. Please log into your account, locate the manuscript record, and check for the action link "View Attachments". If this link does not appear, there are no attachment files.]

To ensure your figures meet our technical requirements, please review our figure guidelines: https://journals.plos.org/plosone/s/figures

You may also use PLOS’s free figure tool, NAAS, to help you prepare publication quality figures: https://journals.plos.org/plosone/s/figures#loc-tools-for-figure-preparation.

NAAS will assess whether your figures meet our technical requirements by comparing each figure against our figure specifications.

# Author comment: Author response to Decision Letter 1

**DOI:** 10.1371/journal.pone.0350926.r002

21 Apr 2026

Reviewer #1

In my view, the framing and international relevance of the manuscript would benefit from the inclusion—either in the Introduction or in a brief contextual paragraph in the Discussion—of a concise overview of the most recent developments in Europe regarding the legalisation/regulation of assisted suicide (and, where relevant, euthanasia). Even a short summary indicating which countries have introduced regulatory changes in recent years and what key regulatory models are emerging would help readers better situate the German case. In this respect, comparative references such as: https://pubmed.ncbi.nlm.nih.gov/32437262/ (10.1177/0025817220923687), https://pubmed.ncbi.nlm.nih.gov/34330794/ (10.1136/medethics-2021-107523) could be helpful.

-> Thank you very much for your valuable comment on the placement of the German legal situation within the European context, and for the references you provided. The introduction has been updated to include a corresponding explanation, as well as a reference to the legal frameworks surrounding assisted suicide in European countries.

“This trend in Germany towards the liberalisation of assisted dying is in line with developments in other European countries in recent years. Following the long-standing legalisation of assisted suicide in Switzerland and of assisted suicide and euthanasia in the Netherlands and Belgium, Italy (2019), Austria (2020), Spain (2021), and France (2025) have also introduced legislation regulating assisted suicide and, in some cases, euthanasia since 2019.”

A second issue concerns the composition of the sample: only 5 of the 16 interviewees report direct involvement in suicide assistance, whereas the others primarily provide indirect knowledge (through research, observation of the public debate, or related professional roles). Although this limitation is already acknowledged by the authors, I believe it should be handled in a more structured way because it affects the strength of the inferences: describing “how the practice works” is not equivalent to describing “how it is reconstructed/perceived by informed stakeholders.” I would therefore recommend distinguishing more systematically—both in the analysis and in the presentation of findings—between informants with direct versus indirect experience, and clarifying how this difference informed coding, interpretation, and the weight attributed to different strands of evidence.

-> Thank you for this important comment.

All statements in the Results section were reviewed once again to verify who had made them. Any statements made solely by individuals who had not yet been directly involved in assisted suicide were introduced accordingly. This procedure is explained at the beginning of the presentation of the interview findings: “Findings derived exclusively from individuals who had not yet been directly involved in assisted suicide are expressly mentioned.”

In the Discussion section, the absence of experts who had been directly involved in assisted suicide was highlighted in relation to the topics ‘assisted suicide outside of right-to-die organisations’ and ‘non-medical assisted suicide providers’:

“The findings of our study on assisted suicide outside of right-to-die organisations, as well as the scope of practice of non-medical suicide assistants, are based primarily on the accounts of individuals who were not directly involved in assisted suicide. The absence of precision in the findings necessitates further research.”

Finally, while I appreciate the mapping of actors, the Results section currently risks reading primarily as a taxonomy of roles and tasks (as summarised in the code tree) rather than a full field description. The categories identified are plausible (organisations, assistants, treating physicians, funeral services, family members, and indirect actors), but it remains less clear how the field operates in practice: what the typical pathways look like, the temporal sequence in which different actors become involved, the minimum steps in the process (from request to death and subsequent procedures), and where the main variants or decision points lie (for instance, differences between organisation-mediated pathways and non-affiliated routes, or the possible involvement of institutional actors). Even a brief, schematic representation of pathways (e.g., as a box or figure) would substantially enhance clarity and added value.

-> Thank you very much for this interesting suggestion. Unfortunately, it is not possible to provide a detailed description of individual pathways based on the expert interviews, but this will be done in the next phase of the research project.

A schematic overview of the assisted suicide process has now been included.

In this context, the distinction between “providers” and “participants” could also be made more consistent throughout the text and the code tree by more clearly specifying the operational criteria and the positioning of indirect actors; this would help to better substantiate certain statements in the Discussion (e.g., “increasing complexity/heterogeneity”), which currently appear more asserted than demonstrated in a structured way.

-> In the code tree, the term “indirect involved providers and participants” was replaced by the term “indirectly involved players” as it is used in the Results section.

In the Results section, the term “indirectly involved players” is concretised:

“Indirectly involved players do not generally support assisted suicides, but may in some cases and under certain circumstances be involved in the process.”

In the Discussion Section, the term “indirectly involved players” was used consistently, and a passage that could have been misinterpreted was clarified:

“Treating physicians, relatives and even funeral directors may occasionally facilitate assisted suicide, while numerous other players appear to be indirectly involved.”

Given the sensitivity of the topic, I also recommend consistent terminology (e.g., using “right-to-die organisations” uniformly), avoiding non-equivalent terms (such as “euthanasia organisations”), and clearly defining the key terms adopted.

-> The term ‘euthanasia organisations’ has been consistently replaced by the term ‘right-to-die organisations’.

Reviewer #2

My main concern is the Data Availability Statement. As written, the statement that data are available from the corresponding author upon reasonable request does not appear sufficient for PLOS ONE. If full public sharing is not possible because of ethical or legal concerns, the manuscript should clearly explain those restrictions and provide a journal-compliant restricted-access pathway.

-> The raw data related to this publication cannot be openly released, as some restrictions due data protection regulations apply. The data contain transcripts of interviews and no interviewees consented to having the complete transcription of their interview shared.

This limitation has been added in the Data Availability section.

Second, the analytic framing should be more precise. The manuscript is strongest as a structured expert-based field description. Because the interview guide was substantially literature-informed and included prompts about specific actors, the findings should not be presented as if the actor categories emerged purely inductively. The authors should frame the paper more explicitly as a qualitative field-mapping study.

-> Thank you very much for this important point. The coding process relating to the providers and participants has been clarified in the Methods section.

“Some categories of providers and participants were derived deductively from the literature (e.g. right-to-die organisations, physicians, relatives and friends, nurses), whilst others were developed inductively from the data. In some cases, deductive categories were refined on the basis of the data (e.g. treating physicians and physician suicide assistants).”

Third, several conclusions should be stated more cautiously. Fewer than half of participants had direct involvement in assisted suicide, and much of the evidence reflects expert knowledge rather than direct observation. Accordingly, claims about practice in Germany should more often be phrased as what interviewees described or what the findings suggest.

-> Thank you for this important comment.

All statements in the Results section were reviewed once again to verify who had made them. Any statements made solely by individuals who had not yet been directly involved in assisted suicide were introduced accordingly.

This procedure is explained at the beginning of the presentation of the interview findings:

“Findings derived exclusively from individuals who had not yet been directly involved in assisted suicide are expressly mentioned.”

In the Discussion section, the absence of experts who had been directly involved in assisted suicide was highlighted in relation to the topics ‘assisted suicide outside of right-to-die organisations’ and ‘non-medical assisted suicide providers’.

“The findings of our study on assisted suicide outside of right-to-die organisations, as well as the scope of practice of non-medical suicide assistants, are based primarily on the accounts of individuals who were not directly involved in assisted suicide. The absence of precision in the findings necessitates further research.”

The Methods section would also benefit from fuller reporting of qualitative rigor, especially sample adequacy, coding review, handling of disagreements, and reflexivity.

-> The Methods section was supplemented:

Sampling

“The interview study was concluded once all identified interviewees had been interviewed and no further interviews revealed any additional providers, participants or interviewees.”

Data analysis

“Some categories of providers and participants were derived deductively from the literature (e.g. right-to-die organisations, physicians, relatives and friends, nurses), whilst others were developed inductively from the data. In some cases, deductive categories were refined on the basis of the data (e.g. treating physicians and physician suicide assistants).”

In addition, terminology should be standardized

-> The term ‘euthanasia organisations’ has been consistently replaced by the term ‘right-to-die organisations’.

the limitations section should more clearly acknowledge likely underrepresentation of less visible or informal practices.

-> The Limitations section has been supplemented:

“The under-representation of interviewees who were directly involved in assisted suicide may also indicate that informal practices in assisted suicide are rarely reported.”

Reviewer #3

I would suggest that the abstract in question would benefit from a better form of framing of the context of the study. This would allow a better understanding of the importance of the topic. Your conclusions should also be improved.

->Due to the limited character count in the abstract, the Background section could not be expanded. However, in the introduction of the main manuscript, the German context is now more firmly situated within the broader European context.

Keywords: are these keywords Mesh terms?

-> Mesh terms were revised and the term “Death, Assisted,” was corrected.

As far as I can see, the literature addressed has not been described accurately. The overall organization and clarity throughout the manuscript should be improved. For the introduction, a restructuring of the writing to provide more coherent and connected ideas and sections would be valuable. A brief synopsis or synthesis of ideas and relationships between or within constructs would improve flow dramatically. It is not clear what knowledge this paper adds from an international perspective. How is this/your study increasing our understanding/knowledge?

->

Thank you very much for your valuable comment regarding the placement of the German legal situation within the European context. A corresponding explanation has been included in the introduction.

“This trend in Germany towards the liberalisation of assisted dying is in line with developments in other European countries in recent years. Following the long-standing legalisation of assisted suicide in Switzerland and of assisted suicide and euthanasia in the Netherlands and Belgium, Italy (2019), Austria (2020), Spain (2021), and France (2025) have also introduced legislation regulating assisted suicide and, in some cases, euthanasia since 2019.”

Please provide the specific questions under study, as it would be helpful for the readers.

-> The research question has been added in the study aim section:

"The research question is: Which providers and participants of assisted suicides in Germany exist and which tasks do they perform?"

The ethical and consent elements should be moved to the end of the Methods section.

-> The ethical and consent elements have been moved to the end of the Methods section.

More information about the study approach, along with its epistemological underpinnings, is, therefore, needed. This needs to be expanded, clarified, and supported by in-text citations. Authors need to pay close attention to the requirements of reporting guidelines for qualitative research (e.g., COREQ checklist).

-> In the design section it was added, that the study followed an interpretative approach.

“The ASEP-study was a prospective, observational qualitative investigation that explored and analysed the practice of assisted suicide in Germany following an interpretative approach (33).”

Missing steps of the COREQ guideline have been added (Items 6-8, 23, 28).

The authors do not follow all the steps of COREQ, such as the presentation of the characteristics of the research context, what kind of relationship the researcher has with the participants, and how the participants were recruited.

-> Missing steps of the COREQ guideline have been added (Items 6-8, 23, 28).

Interviews: Interview guide was it pilot-tested? Were repeat interviews carried out?

-> As described in the Methods section two pilot interviews were conducted.

As no repeat interviews were conducted, this was not reported.

What strategies were developed in the interviews to create rapport with the participants and deal with communication impasses?

-> Information was added in the Data Collection section:

“The interviews commenced with an introductory question. This enabled the participants to introduce themselves and establish a rapport with the interviewer. If an interviewee did not understand a question, it was repeated using different words. If an interviewee did not wish to answer a question, it was omitted.”

Were transcripts returned to participants for comment and/or correction?

-> Information was added at the end of the Data Collection section:

“None of the participants requested to have their transcripts returned. Participants were not asked to comment on the results of the study.”

It is unclear how the authors will ensure the reliability of qualitative coding. Provide this information.

-> We state in the Data Analysis section, the reliability of the qualitative coding was reviewed by a second member of the research team.

The process of analysis should be made as transparent as possible (notwithstanding the conceptual and theoretical creativity that typically characterizes qualitative research). Did participants provide feedback on the findings (triangulation)? How do you ensure methodological rigour? The researcher’s own position should clearly be stated. For example, have they examined their own role, possible bias, and influence on the research (reflexivity)? What experience or training did the researchers have? Did they use reflexive bracketing to achieve openness?

-> The process of data collection and data analysis has been concretised:

Data collection

“None of the participants requested to have their transcripts returned. Participants were not asked to comment on the results of the study.”

Data analysis

“Some categories of providers and participants were derived deductively from the literature (e.g. right-to-die organisations, physicians, relatives and friends, nurses), whilst others were developed inductively from the data. In some cases, deductive categories were

Attachment Submitted filename: Response to Reviewers.docx

# Peer review: Decision Letter 1

**DOI:** 10.1371/journal.pone.0350926.r003

**Authors:** Andrea Cioffi

20 May 2026

Providers of and participants in suicide assistance in Germany: A field description based on expert interviews

PONE-D-25-56777R1

Dear Dr. Schwabe,

We’re pleased to inform you that your manuscript has been judged scientifically suitable for publication and will be formally accepted for publication once it meets all outstanding technical requirements.

Within one week, you’ll receive an e-mail detailing the required amendments. When these have been addressed, you’ll receive a formal acceptance letter and your manuscript will be scheduled for publication.

An invoice will be generated when your article is formally accepted. Please note, if your institution has a publishing partnership with PLOS and your article meets the relevant criteria, all or part of your publication costs will be covered. Please make sure your user information is up-to-date by logging into Editorial Manager at Editorial Manager® and clicking the ‘Update My Information' link at the top of the page. For questions related to billing, please contact billing support.

If your institution or institutions have a press office, please notify them about your upcoming paper to help maximize its impact. If they’ll be preparing press materials, please inform our press team as soon as possible -- no later than 48 hours after receiving the formal acceptance. Your manuscript will remain under strict press embargo until 2 pm Eastern Time on the date of publication. For more information, please contact onepress@plos.org.

Kind regards,

Andrea Cioffi

Academic Editor

PLOS One

Additional Editor Comments (optional):

Reviewers' comments:

Reviewer's Responses to Questions

**Comments to the Author**

1. If the authors have adequately addressed your comments raised in a previous round of review and you feel that this manuscript is now acceptable for publication, you may indicate that here to bypass the “Comments to the Author” section, enter your conflict of interest statement in the “Confidential to Editor” section, and submit your "Accept" recommendation.

Reviewer #1: All comments have been addressed

Reviewer #3: All comments have been addressed

**********

2. Is the manuscript technically sound, and do the data support the conclusions?

The manuscript must describe a technically sound piece of scientific research with data that supports the conclusions. Experiments must have been conducted rigorously, with appropriate controls, replication, and sample sizes. The conclusions must be drawn appropriately based on the data presented.

Reviewer #1: Yes

Reviewer #3: Yes

**********

3. Has the statistical analysis been performed appropriately and rigorously?

Reviewer #1: I Don't Know

Reviewer #3: N/A

**********

4. Have the authors made all data underlying the findings in their manuscript fully available?

The PLOS Data policy requires authors to make all data underlying the findings described in their manuscript fully available without restriction, with rare exception (please refer to the Data Availability Statement in the manuscript PDF file). The data should be provided as part of the manuscript or its supporting information, or deposited to a public repository. For example, in addition to summary statistics, the data points behind means, medians and variance measures should be available. If there are restrictions on publicly sharing data—e.g. participant privacy or use of data from a third party—those must be specified.

Reviewer #1: Yes

Reviewer #3: Yes

**********

5. Is the manuscript presented in an intelligible fashion and written in standard English?

PLOS ONE does not copyedit accepted manuscripts, so the language in submitted articles must be clear, correct, and unambiguous. Any typographical or grammatical errors should be corrected at revision, so please note any specific errors here.

Reviewer #1: Yes

Reviewer #3: Yes

**********

6. Review Comments to the Author

Please use the space provided to explain your answers to the questions above. You may also include additional comments for the author, including concerns about dual publication, research ethics, or publication ethics. (Please upload your review as an attachment if it exceeds 20,000 characters)

Reviewer #1: I thank the Authors for their careful and point-by-point response to my previous comments.

In my view, the revised manuscript has adequately addressed the concerns raised during the review. The authors have clarified the relevant methodological and interpretative issues, and the manuscript has improved substantially as a result.

I therefore consider the manuscript suitable for publication in its revised form.

I would also like to thank the Editor for the opportunity to re-review this work.

Reviewer #3: I believe that the review carried out has greatly improved the quality of the study. Also, I do think that the author(s) address the broad questions that were asked appropriately.

**********

7. PLOS authors have the option to publish the peer review history of their article (what does this mean?). If published, this will include your full peer review and any attached files.

If you choose “no”, your identity will remain anonymous but your review may still be made public.

**Do you want your identity to be public for this peer review?**  For information about this choice, including consent withdrawal, please see our Privacy Policy.

Reviewer #1: No

Reviewer #3: **Yes:** Carlos Laranjeira

**********

# Editor report: Acceptance letter

**DOI:** 10.1371/journal.pone.0350926.r004

**Authors:** Andrea Cioffi

PONE-D-25-56777R1

PLOS One

Dear Dr. Schwabe,

I'm pleased to inform you that your manuscript has been deemed suitable for publication in PLOS One. Congratulations! Your manuscript is now being handed over to our production team.

At this stage, our production department will prepare your paper for publication. This includes ensuring the following:

* All references, tables, and figures are properly cited

* All relevant supporting information is included in the manuscript submission,

* There are no issues that prevent the paper from being properly typeset

You will receive further instructions from the production team, including instructions on how to review your proof when it is ready. Please keep in mind that we are working through a large volume of accepted articles, so please give us a few days to review your paper and let you know the next and final steps.

Lastly, if your institution or institutions have a press office, please let them know about your upcoming paper now to help maximize its impact. If they'll be preparing press materials, please inform our press team within the next 48 hours. Your manuscript will remain under strict press embargo until 2 pm Eastern Time on the date of publication. For more information, please contact onepress@plos.org.

You will receive an invoice from PLOS for your publication fee after your manuscript has reached the completed accept phase. If you receive an email requesting payment before acceptance or for any other service, this may be a phishing scheme. Learn how to identify phishing emails and protect your accounts at https://explore.plos.org/phishing.

If we can help with anything else, please email us at customercare@plos.org.

Thank you for submitting your work to PLOS ONE and supporting open access.

Kind regards,

PLOS ONE Editorial Office Staff

on behalf of

Dr. Andrea Cioffi

Academic Editor

PLOS One
